# microRNA alterations in ALDH positive mammary epithelial cells: a crucial contributing factor towards breast cancer risk reduction in case of early pregnancy

**DOI:** 10.1186/1471-2407-14-644

**Published:** 2014-08-31

**Authors:** Sushmita Bose Nandy, Ramadevi Subramani, Venkatesh Rajamanickam, Rebecca Lopez-Valdez, Arunkumar Arumugam, Thiyagarajan Boopalan, Rajkumar Lakshmanaswamy

**Affiliations:** Department of Biomedical Sciences MSB1, Center of Excellence in Cancer Research, Paul L. Foster School of Medicine, Texas Tech University Health Sciences Center, 5001 El Paso Drive, El Paso, TX 79905 USA

**Keywords:** Pregnancy, ALDH positive MECs, Breast cancer, microRNAs

## Abstract

**Background:**

microRNAs have recently succeeded in grabbing the center stage in cancer research for their potential to regulate vital cellular process like cell cycle, stem cell renewal and epithelial mesenchymal transition. Breast cancer is the second most leading cause of cancer related mortality in women. The main reason for mortality is chemoresistance and metastasis for which remnant stem cells are believed to be the cause. One of the natural ways to reduce the risk of breast cancer in women is early pregnancy. Unraveling the mechanism behind it would add to our knowledge and help in evolving newer paradigms for breast cancer prevention.

The current study deals with investigating transcriptomic differences in putative stem cells in mammary epithelial cell population (MECs) in terms of genes and microRNAs. *In silico* tools were used to identify potential mechanisms. ALDH positive MECs represent a putative stem cell population in the mammary gland.

**Methods:**

MECs were extracted from the mammary gland of virgin and parous (one time pregnant) rats. ALDH positive MECs were sorted and used for transcriptional and translational analysis for genes and microRNAs. *In silico* analysis for target prediction and networking was performed through online portals of Target Scan and Metacore.

**Results:**

A total of 35 and 49 genes and microRNAs respectively were found to be differentially expressed within the two groups. Among the important genes were Lifr, Acvr1c, and Pparγ which were found to be targeted by microRNAs in our dataset like miR-143, miR-30, miR-140, miR-27b, miR-125a, miR-128ab, miR-342, miR-26ab, miR-181, miR-150, miR-23ab and miR-425. *In silico* data mining and networking also demonstrates that genes and microRNA interaction can have profound effects on stem cell renewal, cell cycle dynamics and EMT processes of the MEC population.

**Conclusions:**

Our data clearly shows that certain microRNAs play crucial role in the regulation of ALDH positive MECs and favor an anti-carcinogenic environment in the post-partum gland. Some of the potential interplaying mechanisms in the ALDH positive MEC population identified through this study are p21, Lifr and Pparγ mediated cell cycle regulation, regulation of metastasis and expansion of stem cell pool respectively.

**Electronic supplementary material:**

The online version of this article (doi:10.1186/1471-2407-14-644) contains supplementary material, which is available to authorized users.

## Background

Breast cancer is one of the primary causes of cancer-related deaths and the most common malignancy in women worldwide [1]. Despite different existing and potential strategies to treat breast cancer, its global incidence is predicted to increase in the coming years at a rate of 3.2 million new cases every year by 2050 [2]. Currently, while there are good treatment options for patients with breast cancer, a high percentage of patients develop resistance to these treatments over time, and these therapies often have undesirable and harmful side effects.

It is well known that early, full-term pregnancy reduces the risk of breast cancer. A completed full-term pregnancy before the age of 20 years reduces breast cancer risk by 50% compared to nulliparous women [3]. While this fact has been known for a long time, different reasons have been brought forward repeatedly to explain this phenomenon. Unfortunately, the mechanism behind this protective phenomenon is not well defined. One possible explanation is that parous women often have a different hormonal profile compared to nulliparous women [4]. Thus, it is thought that alterations in the hormonal milieu both during and after pregnancy may contribute to the phenomenon of parity-induced protection against breast cancer. Animal studies have proven that short-term treatment with pregnancy levels of estrogen can be effective in reducing mammary cancer incidence [5, 6]. Also of special interest is the hormone prolactin, which has been found at reduced levels in the sera of parous women; coincidentally, prolactin-suppressing drugs have been shown to reduce mammary tumors [7]. Further, growth hormone has also been demonstrated to be vital for breast cancer development and parity reduces the levels of growth hormone in circulation [8–10]. Thus, strong evidence suggests a definitive role for hormones in parity-induced protection against breast cancer. Further, some researchers have suggested that pregnancy results in the terminal differentiation of the mammary gland, resulting in the loss of a particular cell population that is prone to malignant transformation [11, 12]. However, other studies indicate that differentiation of the mammary gland *per se* is not sufficient to explain the phenomenon of parity-induced protection against breast cancer [5, 6, 13].

It has been well established that the mammary gland is partly comprised of a population of epithelial stem cells that are capable of self-renewal and are responsible for the generation of newer cell types specific to the gland. Therefore, a third theory was proposed that breast cancer arises primarily from the stem cell compartment and pregnancy may lead to protective changes in the stem cell population of the mammary gland. However, it remains highly debatable whether the mammary epithelial stem cell population is a primary contributing factor to the phenomenon of parity-induced protection [14–17], and additional work in this area is therefore needed. A recent report by Siwko *et al.* [14] suggested that there is a persistent decrease in the number of mammary-repopulating units (mammary epithelial stem cells) after parity. In contrast, there are reports, including ours, which demonstrate that parity-induced protection is not due to changes in the number of cells in the mammary epithelium itself but is the result of systemic changes in the whole organism [18–20]. Thus, it is imperative to understand how the systemic environment influences mammary epithelial stem cells and how this may contribute to the protective phenomenon of parity. The significance of this study lies in the notion that stem cells are the initiators of carcinogenesis, according to the cancer stem cell theory.

Over the last couple of years, research in the field of breast cancer, has added strong lines of evidence, supporting the fact that microRNAs have a significant role to play in the regulation of the signaling pathways involved in oncogenesis. They have also been implicated in the maintenance of cancer stem cells via their ability to affect multiple pathways including cell proliferation, cell death [21–23], cell- cell communication and cell adhesion [24].

In this study, we demonstrate that pregnancy alters molecular processes in ALDH positive MECs (putative mammary epithelial stem cells), leading to a decreased risk of mammary cancer. Here, we primarily focused on the genetic differences of ALDH positive MECs from both virgin and parous animals, through gene and microRNA profiling. To the best of our knowledge, this is the first study to identify the parity-induced microRNA signature in the ALDH positive MECs that is associated with the reduced risk of breast cancer.

## Methods

### Animals

Virgin Lewis rats were purchased from Harlan Sprague–Dawley (Indianapolis and San Diego). The rats were housed in temperature controlled room with 12-h light/dark schedule. They were fed (Teklad 8640; Teklad, Madison, WI) and water ad libitum. To generate parous animals, seven week old virgin rats were mated with similar aged male rats. The pups were removed from the cage right after parturition. The mammary glands from the parous rats were removed six weeks after parturition to allow involution of the gland. Mammary glands were removed from age-matched virgin rats and used as controls. All procedures performed were approved and conducted in accordance with the Texas Tech University Health Sciences Center Institutional Animal Care and Use Committee’s guidelines.

### Mammary epithelial cell isolation and stem cell enrichment

Isolation of MECs from the mammary gland was carried out using collagenase assisted cell dissociation. Briefly, all six pairs of mammary glands from both virigin and uniparous (one-time pregnant) Lewis rats were processed by mechanical and enzymatic dissociation to prepare a single-cell suspension. MECs were cultured overnight. They were then stained with ALDEFLUOR (Stem Cell Technologies), which enabled the selection of putative stem cells from MECs with strong aldehyde dehydrogenase (ALDH) activity. An inhibitor of ALDH was used as the negative control. The cells were analyzed on a flow cytometer in the green fluorescence channel (520–540 nm) and were then subjected to sorting. Sorting was performed using a flow cytometric cell sorter (BD FACS Aria) to collect the ALDH^bright^ population. These sorted ALDH positive MECs were then used to prepare RNA and protein lysates for transcriptional and translational analysis, respectively.

### Mammosphere assay

The mammosphere formation assay was performed as previously described [25]. Briefly, MECs from both virgin and parous animals were plated after preparation of a single-cell suspension using a 23-G needle. Cells were plated in ultralow attachment 6-well plates (Corning) with mammosphere media containing B27 supplement and LONZA Single Quot supplements (hydrocortisone, insulin, beta-mercaptoethanol, EGF, and gentamycin) in phenol red-free DMEM/F12 media (GIBCO). The cell density of this assay was optimized to 500 cells/cm^2^. The cells were not disturbed for 5 days before any change in media. After 7 days, any sphere larger than 50 μm was considered for counting and further analysis, using a sample size of six.

### Immunofluorescence

The mammospheres were collected by centrifugation at 115 × *g* for 5 min and were gently suspended in 200 μl mammosphere media. They were then plated on poly-lysine coated; 8-well chambered slides with mammosphere assay containing 1% fetal bovine serum and incubated at 37°C, with 5% CO_2_ for 3–4 hrs for attachment. These mammospheres were then fixed using 5% formaldehyde and blocked with 5% bovine serum albumin for 1 hr. They were then stained for stem cell markers using primary antibodies against SOX2 (goat IgG clone Y-17, 1:100 dilutions, Santa Cruz Biotechnology) and OCT3/4 (mouse IgG2b clone C-10, 1:50 dilution, Santa Cruz Biotechnology). Alexafluor 488 and 594 were used as secondary antibodies raised in species appropriate for the primary antibody. The spheres were washed and counterstained with DAPI and mounted. All slides were examined using a Nikon confocal microscope (Eclipse Ti, Nikon, Japan). Multicolor images were collected sequentially in three channels.

### Proliferation assay

An EdU (5-ethnyl-2′-deoxyuridine) based kit; Click-iT EdU Imaging kit was used to perform the assay (Molecular probes, Life technologies). The sorted ALDH positive MECs were plated in the 8 well chamber slide with 1 × 10^4^ cells/ well and incubated overnight at 37°C/ 5% CO_2._ 10 μM of EdU was incubated with the cells for 2 hrs at 37°C/5% CO_2_. The cells were then fixed with 3.7% formaldehyde for 15 min and permeabilized with 0.5% Triton-X-100 for 20 min. It was then incubated with Alexa fluor azide for 30 min to enable the detection of EdU. They were finally counterstained with DAPI and mounted for examination using a Nikon confocal microscope (Eclipse Ti, Nikon, Japan).

### Gene and microRNA arrays and *in silico*analysis

Gene and global microRNA profiles were generated using the SABiosciences PCR (Cat No. PARN-405Z and PARN-047Z) and miRNome array (Cat No. MIRN-216Z), respectively. Briefly, RNA was extracted from ALDHpositive MECs of both virgin and parous animals using Trizol (Invitrogen). Replicates of mammary tissues from at least six animals from each group were used for gene and miRNome array analyses. For the gene array, 160 genes associated with stemness and stem cell development were analyzed. For the microRNA array, 653 of the most abundantly expressed and well-characterized microRNAs in the rat microRNA genome as annotated by miRBase Release 16 were profiled. Genes or microRNAs with at least a 2-fold increase or decrease in expression were considered significantly up- or downregulated, respectively.

For microRNA target prediction, the main *in silico* approach used depends on sequence complementarity. To predict the targets for the microRNA array, we used the online portal of TargetScan. The targets in which the paired sites were highly conserved were considered for further analysis. TargetScan (http://www.targetscan.org/) predicts the biological targets of microRNAs by searching for the presence of conserved 8mer and 7mer sites that match the seed region of each microRNA. To increase the signal-to-noise ratio, TargetScan requires strict complementarity between the seed region of the microRNA and the predicted target. TargetScan Human considers matches to annotate human UTRs and their orthologs, as defined by UCSC whole-genome alignments (http://genome.ucsc.edu/). Conserved targeting has also been detected within open reading frames (ORFs). MetaCore from Thomson Reuters was used to perform data mining and pathway analysis for the differentially regulated microRNAs and genes.

### Western blot analysis

Protein lysates of ALDH positive MECs were prepared from glands of virgin and parous animals. Protein concentration was measured using the Pierce BCA (bicinchoninic acid) protein assay (Thermo Scientific) according to the manufacturer’s specifications. Equivalent amounts of protein (1 – 5 μg) were resolved using reducing SDS-PAGE in 4-20% gradient pre-cast Mini-Protean TGX polyacrylamide gels (Bio-Rad). Resolved proteins were transferred onto PVDF immunoblotting membranes and probed with the following antibodies as per manufacturer’s instructions: LIFR, PPAR γ, CYCLIN D, ACVR1C, LIN 28 (Santa Cruz), NOTCH 2, CYCLIN E1, SNAIL, SLUG, VIMENTIN, N-CADHERIN, E-CADHERIN, ZEB (Cell Signaling) and P21 (Abcam). Each membrane was also stripped and reprobed for β-actin as protein loading. For chemiluminescent detection of proteins, SuperSignal West Femto Chemiluminescent Substrate (ThermoScientific) was used according to the manufacturer’s instructions. Immunoblots were processed digitally on the ImageQuant LAS4000 biomolecular imager (GE Healthcare). The signal intensities for each antibody was densitometrically analyzed and normalized to actin bands.

### Statistical analysis

Data are expressed as means with standard deviation or standard error. Student’s *t*-test was used to determine statistical significance between 2 groups. A value of p < 0.05 was considered a statistically significant difference.

## Results

The main aim of this study was to determine the molecular differences in ALDH positive MECs as a result of pregnancy. The model system that we used for this study is FACS-enriched MECs positive for ALDH from virgin and parous animals.

### Parity does not influence the proportion or stemness of ALDH positive MECs

**Figure 1:**
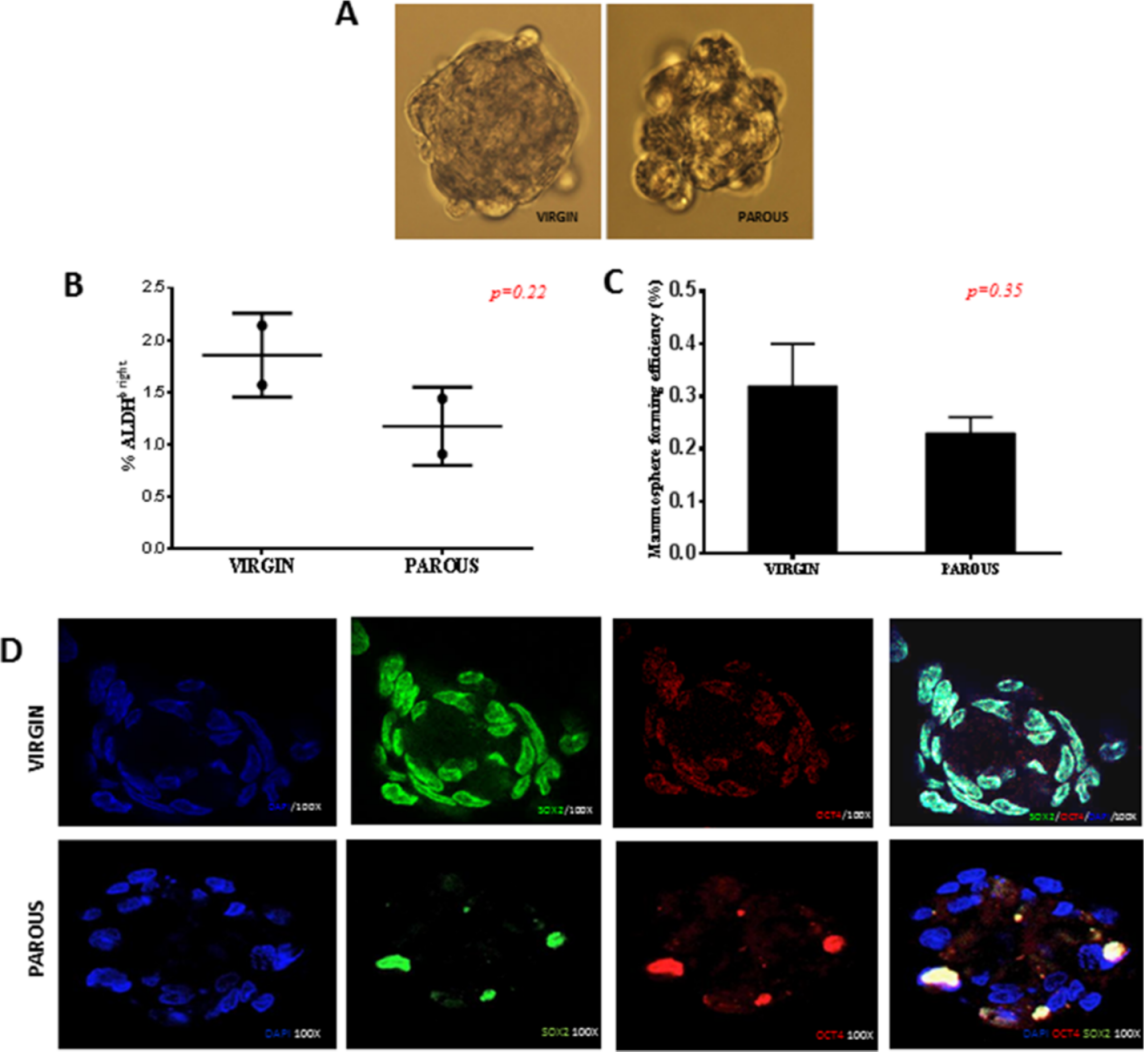
**Quantitative and qualitative analysis of ALDH positive MECs in virgin and parous glands.** Mammosphere formation assay (n = 6) was established from virgin and parous MECs after cell dissociation of the mammary gland. **A)** Mammospheres obtained from virgin and parous rat MECs after 7 days in culture **B)** Percentage of ALDH^bright^ virgin and parous MECs using flow cytometry. The positively stained cells were detected using the green fluorescence channel (520–540 nm) of the flow cytometer. **C)** Mammosphere formation efficiency; total number of mammospheres formed after 7 days in culture were counted and divided by the total number cells initially plated. **D)** Immunofluorescence images showing the presence of stem cell markers like SOX2 and OCT4 in mammospheres obtained from MECs of both the groups. Mammospheres, after 7 days in culture were placed on poly-l-lysine coated chamber slides for attachment for 3-4 hrs and followed by sequential double immunofluorescence staining procedure. The cells from both groups showed positivity for SOX2 and OCT4 indicating the enrichment of stem cells in the mammospheres.

First, we quantified ALDH positive MECs from virgin and parous animals; we did not observe a statistically significant change in the number of ALDH positive MECs between the two groups (*p = 0.22*) (Figure 1A & 1B). Additionally, there were no statistically significant differences in their mammosphere-forming capacities *(p = 0.35)* (Figure 1C). The presence of stem cells in mammospheres derived from virgin and parous MECs was confirmed using stemness markers SOX2 and OCT4 (Figure 1D). Overall, the data indicate that parity does not influence the stem cell population by significantly altering the percentage of ALDH positive MECs compared to a virgin mammary gland. On contrary, our cell proliferation experiment indicated that virgin ALDH positive MECs had a slightly higher proliferation rate than parous ALDH positive MECs (Additional file 1: Figure S1).

### Parity induces alterations in the genetic environment of the ALDH positive MEC population

**Figure 2:**
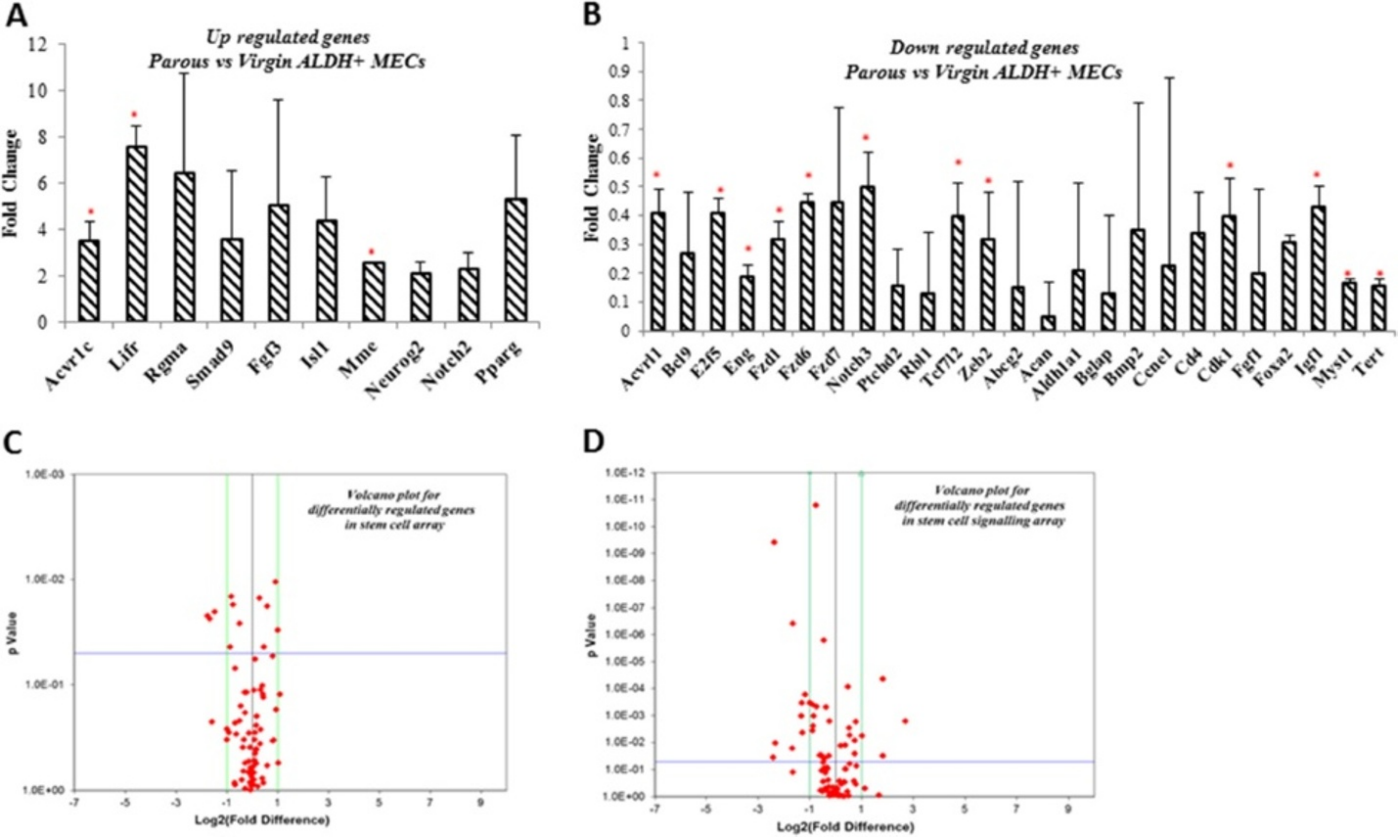
**Pregnancy alters gene expression profile in ALDH** **positive MECs.** Putative stem cells were obtained from both virgin and parous glands by sorting for ALDH^bright^ population. RNA was extracted and cDNA was prepared post DNAase treatment. PCR array was performed (n = 6) to evaluate the expression status of stem cell associated genes in these cells from the two groups. A threshold of 2 folds up- or down-regulation was considered to generate a dataset of the differentially regulated genes between the two groups. **A)** Relative expression of all up regulated genes in ALDH positive MECs of parous rats compared to virgin **B)** Relative expression of all down regulated genes in ALDH positive MECs of parous animals compared to virgin **C)** &**D)** Volcano plot of gene expression between parous and virgin ALDH positive MECs, demonstrating the most significantly differentially expressed genes in two different arrays.

**Figure 3:**
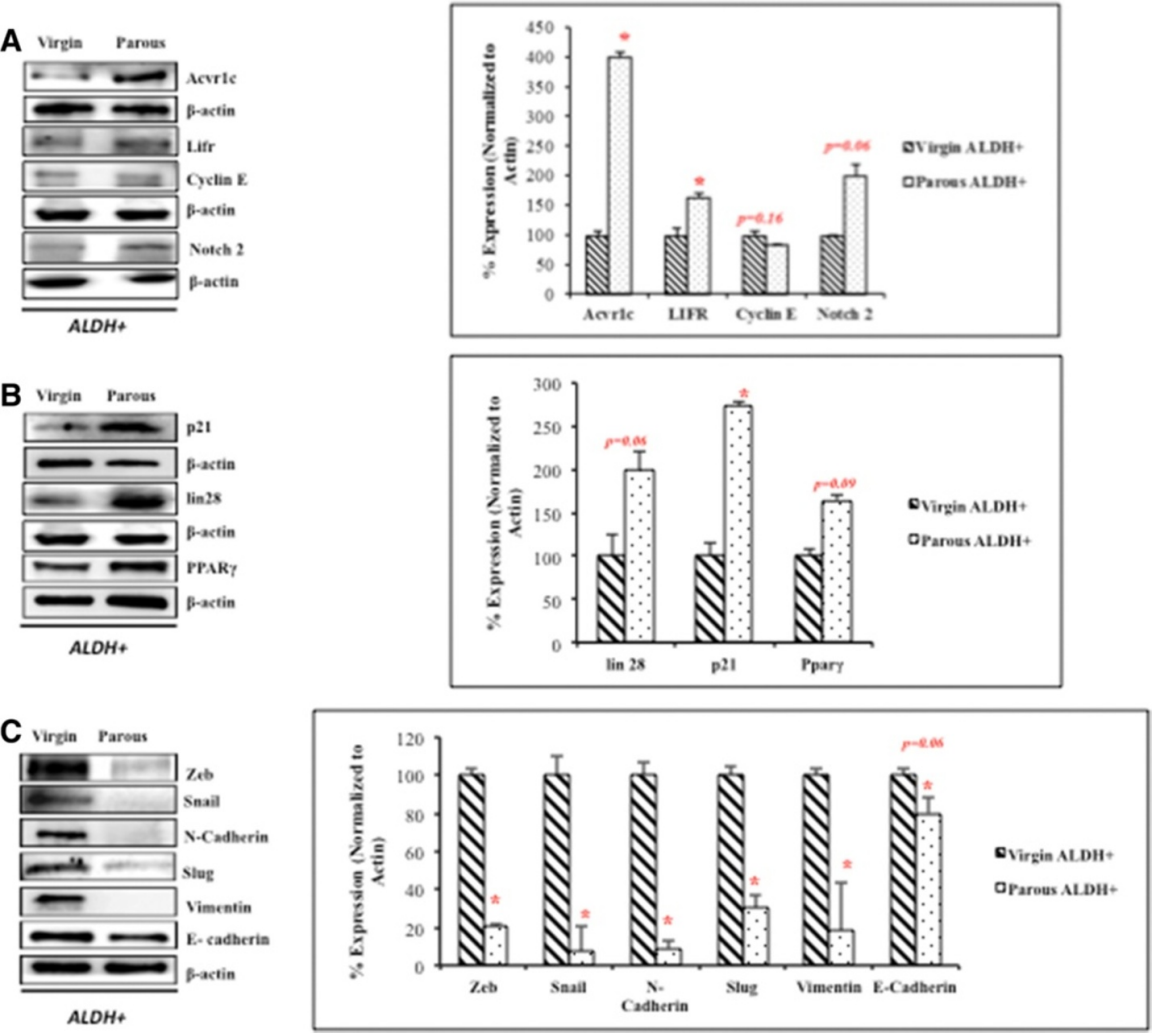
**Regulation of target protein expression by differentially expressed microRNAs in the ALDH positive MECs of parous and virgin animals.** Proteins were extracted from ALDH positive MECs of virgin and parous rat mammary gland. The western blots were repeated atleast thrice for every protein. **A)** &**B)** Immunoblots and corresponding densitometry graphs of proteins (n = 6) for some of the differentially regulated genes e.g. ACVR1C, LIFR, CYCLIN E,NOTCH 2, LIN 28, P21 and PPARγ **C)** Immunoblots and densitometric analysis for EMT related markers like ZEB, SNAIL, N-Cadherin, SLUG, Vimentin and E-Cadherin demonstrate enhanced EMT like characteristics at the translational level in virgin ALDH positive MECs.

We next investigated potential differences in the expression of genes that influence stemness and stem cell properties of ALDH positive MECs in virgin and parous animals. We identified a total of 21% (35/168) of genes that were differentially expressed in parous compared to virgin ALDH positive MECs. We observed that leukemia inhibitory factor receptor (Lifr), RGM domain family member A (Rgma), fibroblast growth factor 3 (Fgf3), and activin A receptor, type IC (Acvr1c) were all strongly upregulated in parous ALDH positive MECs (Figure 2*;* Table 1). Further, Lifr was the most upregulated gene, exhibiting a 7.6-fold increase in parous compared to virgin ALDH positive MECs. We investigated the protein levels of some of these differentially regulated genes, including LIFR, NOTCH2, ACVR1C, p21, CYCLIN D, CYCLIN E1, LIN 28, PPAR γ, SNAIL, SLUG, VIMENTIN, N-CADHERIN, E-CADHERIN and ZEB2. Interestingly, we found the same trend was reflected at the protein level as well (Figure 3). The downregulated genes in parous ALDH positive MECs included the metastasis-promoting gene, Zeb2, cell cycle-associated E2F transcription factor 5 (E2f5) cyclin E1 (Ccne1) and retinoblastoma-like protein 1 (Rbl1) indicating inhibitory activity at the G1–S phase of the cell cycle (Figure 2*;* Table 2).

**Table 1:** **List of genes up regulated in ALDH positive MECs derived from normal parous as compared to virgin mammary gland of rat**

mRNA	Description	GeneBank ID	p- value ( ***t***-test)
**Acvr1c**	Activin A receptor, type IC	NM_139090	*0.000045*
**Lifr**	Leukemia inhibitory factor receptor alpha	NM_031048	*0.0084*
**Rgma**	RGM domain family, member A	NM_001107524	*0.0016*
**Smad9**	SMAD family member 9	NM_138872	*0.03*
**Fgf3**	Fibroblast growth factor 3	NM_130817	*0.123*
**Isl1**	ISL LIM homeobox 1	NM_017339	*0.03*
**Mme**	Membrane metallo-endopeptidase	NM_012608	*0.01*
**Neurog2**	Neurogenin 2	XM_227716	*0.101*
**Notch2**	Notch homolog 2 (Drosophila)	NM_024358	*0.93*
**Pparg**	Peroxisome proliferator-activated receptor gamma	NM_013124	*0.548*

**Table 2:** **List of genes down regulated in mammary epithelial SC derived from normal parous as compared to virgin mammary gland of rat**

mRNA	Description	GeneBank ID	p-value ( ***t***-test)
**Acvrl1**	Activin A receptor type II-like 1	NM_022441	*0.0043*
**Bcl9**	B-cell CLL/lymphoma 9	NM_001107703	*0.122*
**E2f5**	E2F transcription factor 5	XM_574892	*0.0003*
**Eng**	Endoglin	NM_001010968	*0.0001*
**Fzd1**	Frizzled homolog 1 (Drosophila)	NM_021266	*0.02*
**Fzd6**	Frizzled homolog 6 (Drosophila)	NM_001130536	*0.0001*
**Fzd7**	Frizzled homolog 7 (Drosophila)	XM_237191	*0.46*
**Notch3**	Notch homolog 3 (Drosophila)	NM_020087	*0.0003*
**Ptchd2**	Patched domain containing 2	NM_001107992	*0.667*
**Rbl1**	Retinoblastoma-like 1 (p107)	XM_001055763	*0.01*
**Tcf7l2**	Transcription factor 7-like 2 (T-cell specific, HMG-box)	XM_001054844	*0.001*
**Zeb2**	Zinc finger E-box binding homeobox 2	NM_001033701	*0.016*
**Abcg2**	ATP-binding cassette, subfamily G (WHITE), member 2	NM_181381	*0.224*
**Acan**	Aggrecan	NM_022190	*0.292*
**Aldh1a1**	Aldehyde dehydrogenase 1 family, member A1	NM_022407	*0.264*
**Bglap**	Bone gamma-carboxyglutamate (gla) protein	NM_013414	*0.763*
**Bmp2**	Bone morphogenetic protein 2	NM_017178	*0.849*
**Ccne1**	Cyclin E1	NM_001100821	*0.892*
**Cd4**	CD4 molecule	NM_012705	*0.285*
**Cdk1**	Cyclin-dependent kinase 1	NM_019296	*0.043*
**Fgf1**	Fibroblast growth factor 1	NM_012846	*0.333*
**Foxa2**	Forkhead box A2	NM_012743	*0.231*
**Igf1**	Insulin-like growth factor 1	NM_178866	*0.014*
**Myst1**	MYST histone acetyltransferase 1	NM_001017378	*0.023*
**Tert**	Telomerase reverse transcriptase	NM_053423	*0.021*

Several of the differentially regulated genes were found to be involved in cell cycle regulation, stem cell self renewal, and differentiation, including Ccne1, Fgf1, Fgf3, Notch2, Myst histone acetyltransferase 1 (Myst1), Neurogenin 2 (Neurog2), Forkhead box A2 (Foxa2), and ISL Lim homeobox 1 (Isl1). In addition, our stem cell gene array data indicates that TGF-β, NOTCH, and WNT pathways which are involved in stem cell regulation were most influenced by parity. Interestingly, genes associated with identification of stemness, such as Abcg2 and Aldh1a1, were down regulated in parous ALDH positive MECs.

### MicroRNA profiles in virgin and parous ALDH positive MECs

**Figure 4:**
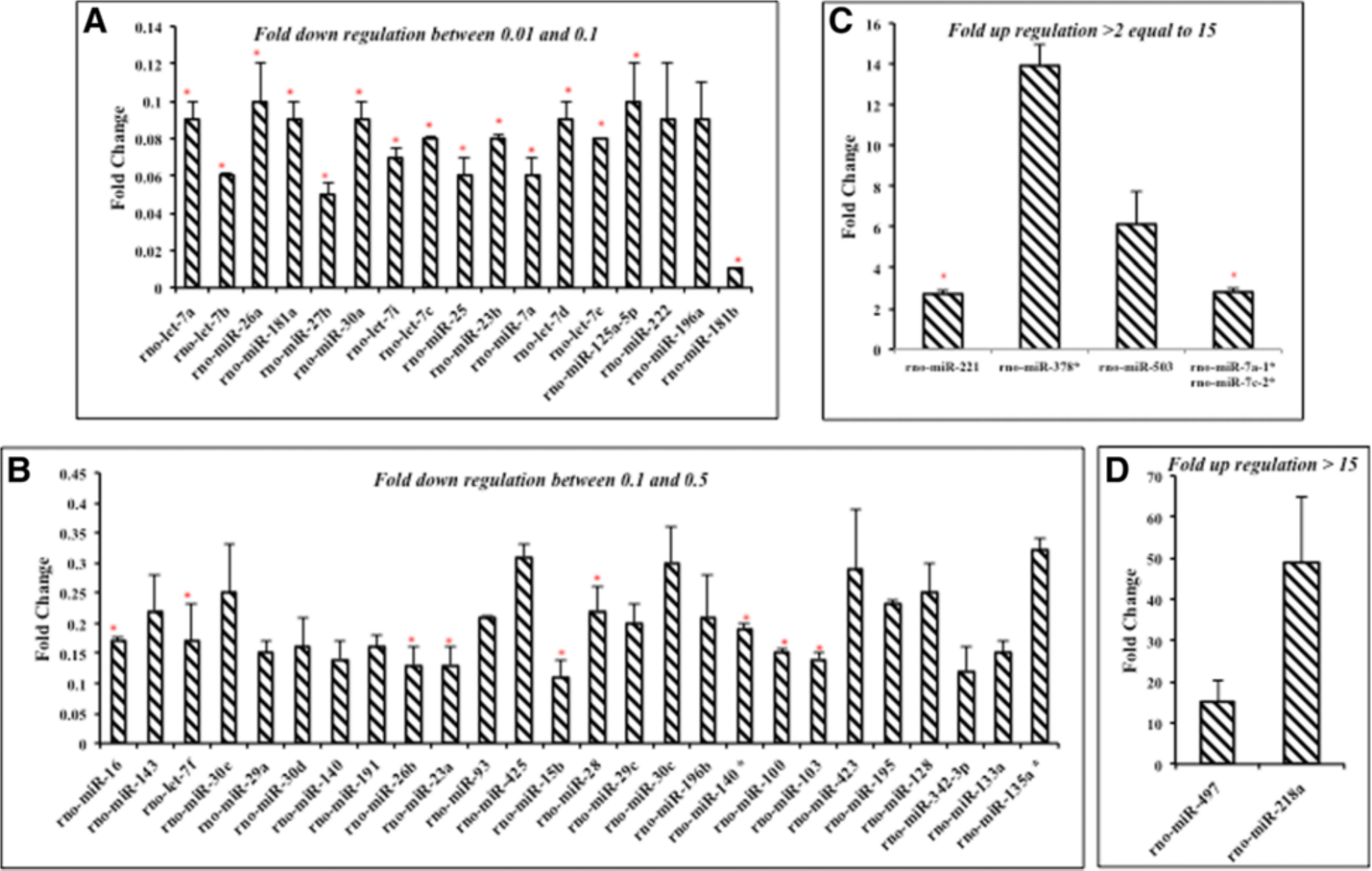
**Whole genome microRNA array revealed the differentially regulated microRNAs among virgin and parous ALDH positive MECs.** Primary rat MECs were obtained after cell dissociation of the mammary gland tissue from virgin and parous animals. The primary cells were subjected to ALDH staining followed by flow cytometric sorting of ALDH^bright^ cell population. RNA was extracted and cDNA (small microRNA specific) was prepared from these cells. The cDNA was then used to perform whole genome microRNA profiling (n = 6) for ALDH positive MECs from both the groups. MicroRNAs that were 2 folds up-or-down-regulated between the groups were considered as differentially regulated. **A)** microRNAs in ALDH positive MECsof parous compared to the virgin glands with a fold down regulation of 0.01 and 0.1. **B)** Sub graph showing the microRNAs which had a fold down regulation between 0.1 and 0.5. This group had the most number of down regulated microRNAs from the dataset. **C)** Up regulated microRNAs in ALDH positive MECs of the parous compared to the virgin glands which had a fold up regulation > 2 through 15. **D)** Sub group of microRNAs which had a fold up regulation of >15.

With the aim of finding novel mechanisms for parity-induced protection against breast cancer, we next investigated global microRNA expression in parous and virgin ALDH positive MECs. The global microRNA profiling data revealed differential regulation of 7.5% (49/653) of the total microRNAs between virigin and parous ALDH positive MECs. Among the differentially regulated microRNAs 12.2% (6/49) and 87.8% (43/49), were up and down regulated respectively; in parous compared to virgin ALDH positive MECs (Figure 4, Table 3 & 4). This screening confirmed the differential expression of several microRNAs in parous ALDH positive MECs that have been reported to be associated with breast and other cancers (Table 5). Many of the downregulated microRNAs in parous ALDH + MECs have been previously reported to be pro-carcinogenic (Table 5). In contrast, a few of the upregulated microRNAs in this case have been previously reported as anti-carcinogenic in nature (Table 5).

**Table 3:** **List of downregulated microRNA in ALDH positive MECs derived from normal parous as compared to virgin mammary gland of rat**

miRNA	PREDICTED TARGET GENES (from gene array)
DOWNREGUALTED	UPREGULATED
rno-miR-143	*LIFR*
rno-let-7a, 7d, 7e, 7b, 7c, 7f, 7i	*ACVR1C, PPARGC1A*
rno-miR-26a, 26b	*ACVR1C*
rno-miR-30a, 30e, 30c, 30d	*LIFR, PPARGC1A, PPARGC1B*
rno-miR-181a, 181b	*ACVR1C, RGMA*
rno-miR-140	*LIFR*
rno-miR-322	*RGMA*
rno-miR-27b	*ACVR1C, ISL1, PPARG, LIFR, SMAD9*
rno-miR-23ab	*ACVR1C, Isl1, PPARG*
rno-miR-93	*RGMA, NEUROG2, PPARGC1B*
rno-miR-425	*ACVR1C*
rno-miR-15b	*RGMA*
rno-miR-25	*PPARGC1B*
rno-miR-125a-5p	*ACVR1C, LIFR*
rno-miR-195	*RGMA*
rno-miR-128 ab	*ACVR1C, ISL1, PPARG, LIFR, SMAD9*
rno-miR-342-3p	*LIFR*
rno-miR-29 ac	*ISL1, NOTCH2*
rno-miR-140-3p	*ACVR1C, PPARGC1A*
rno-miR-103	*PPARGC1A,CCNE1*
rno-miR-423	*PPARGC1B, TCF7L2*

**Table 4:** **List of upregulated microRNA in ALDH positive MECs derived from normal parous as compared to virgin mammary gland of rat**

miRNA	PREDICTED TARGET GENES (from gene array)
UPREGULATED	DOWNREGUALTED
rno-miR-221	*TCF7L2, CD4*
rno-miR-497	*CCNE1, FOXA2*
rno-miR-503	*TCF7L2, BCL9, CCNE1*
rno-miR-218a	*MYST1,IGF1, CCNE1, BCL9, ZEB2*

**Table 5:** **Carcinogenic traits of some of the microRNAs**

miRNA	Carcinogenic trait	References
rno-miR-23b	*Pro-carcinogenic*	L Jin, 2013 [35]
rno-miR-27b	*Pro-carcinogenic*	Wang, 2009 [36]
rno-miR-23a	*Pro-carcinogenic*	L Bhushan, 2011 [37]
rno-miR-93	*Pro-carcinogenic*	Liu S, 2009 [38]
rno-miR-103	*Pro-carcinogenic*	Chen HY, 2012 [39]
rno-miR-423	*Pro-carcinogenic*	Farazi TA, 2011 [40]
rno-miR-195	*Pro-carcinogenic*	Heneghan HM, 2010 [41]
rno-miR-196a	*Pro-carcinogenic*	Jedlinski DJ, 2011 [42]
rno-miR-342-3p	*Pro-carcinogenic*	Savad S, 2012 [43]
rno-miR-135a	*Pro-carcinogenic*	Y Chen, 2012 [44]
rno-miR-497	*Anti-carcinogenic*	Shen L, 2012 [45]

miR-497, miR-218a, miR-378*, miR-503, miR-7a/7c and miR-221 were upregulated in parous ALDH positive MECs. Most of these microRNAs have been shown to have tumor suppressive properties in breast and other cancers. miR-497, miR-218a, miR-378 and miR-503 were reported to have anti-carcinogenic effect in breast, glioma, liver, endometrial and gastric cancers respectively. These microRNAs suppress tumor growth by affecting cell proliferation, stem cell renewal, angiogenesis and cancer cell metabolism [26–31]. miR-221 has been shown to be involved in the promotion of epithelial-mesenchymal transition (EMT) in breast cancer cell lines, where it is regulated by Slug [32]. However, in normal human umbilical vein endothelial cells, miR-221 has been shown to directly target and repress Zeb2 [33].

Both miR-27b and miR-181b were among the most downregulated microRNAs in parous compared to nulliparous ALDH positive MECs. Positive expressions of both these microRNAs have been correlated with poor clinical outcomes as they target genes associated with tumor suppression and neoplastic transformation (high mobility group A proteins) [34, 35].

Thus, the microRNA prolife of parous ALDH positive MECs appears to favor anti-carcinogenesis, at least considering the differentially expressed microRNAs observed in this study. However, functional studies to further support this statement are warranted.

### *In silico*analysis reveals interconnections between differentially regulated microRNAs and genes

**Figure 5:**
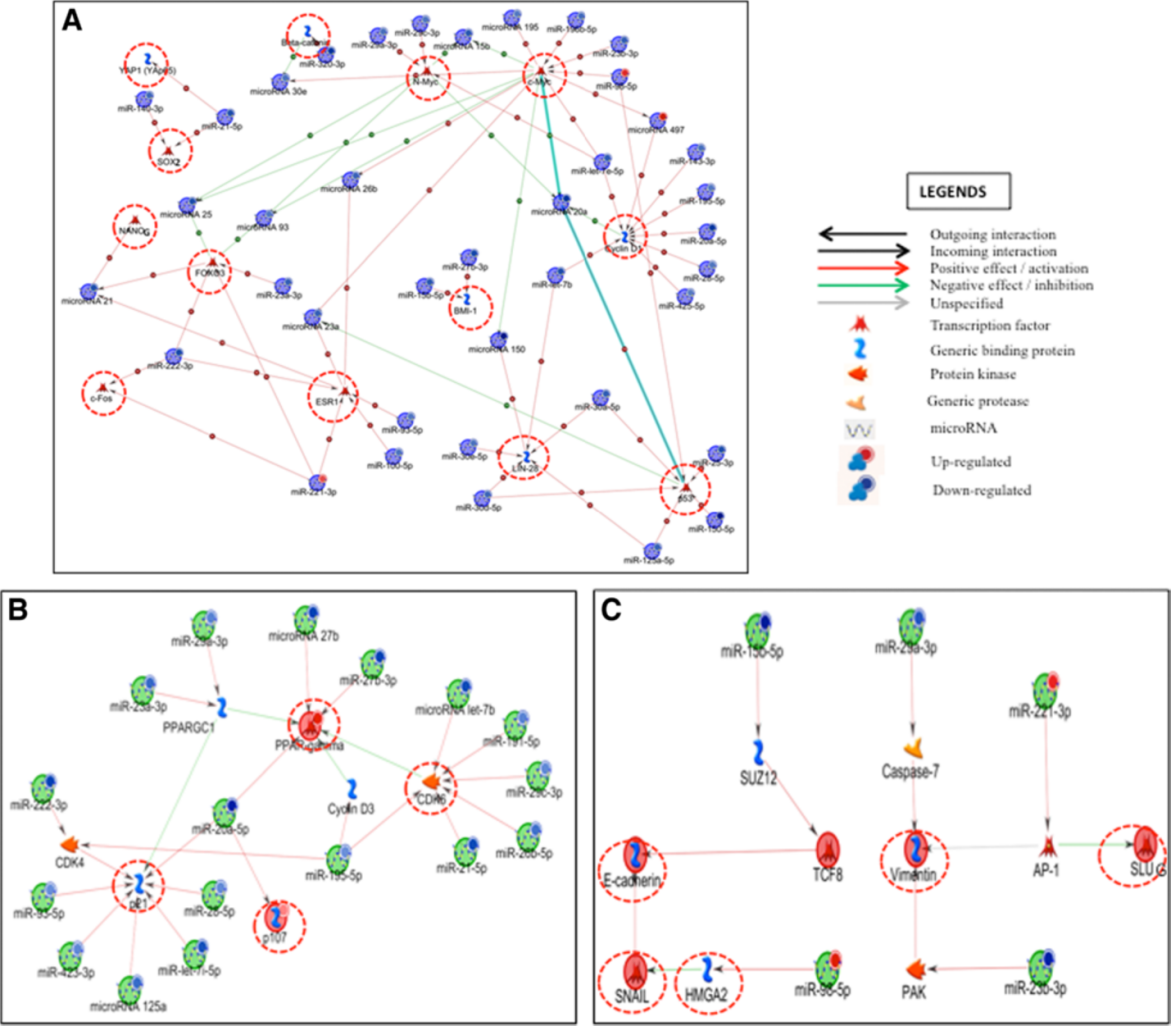
**In silico network analysis describing interactions between differentially regulated microRNAs and some of the relevant proteins.** Gene and microRNA datasets were analysed using Metacore from Thomson Reuters to demonstrate existing interactions between various components of the data sets. **A)** Descriptive network analyzing the microRNA dataset demonstrating the potential molecular interactions among microRNAs with many of the genes/proteins associated with stem cell or tumor suppressive/promoting functions. microRNAs in the dataset were found to be interacting with stem cell associated genes/proteins like BMI-1, LIN-28, SOX2 and NANOG. It was also observed that many of the microRNAs play a role in the regulation of proto-oncogenes like c-fos and c- and n-Myc or tumor suppressor like FOXO3 and p53. The interactions were found to be both direct and indirect in nature. **B)** Network analysis for the microRNAs to visualize their interactions with proteins involved in cell cycle regulation like P21, RBL1 (p107), PPARγ and CDKs. The interaction and expression levels of microRNAs in the parous compared to virgin ALDH positive MECs indicates that there possibly can be increased expression of P21 in the stem cells of the former group. **C)** Network depicting the possible regulation of EMT related markers like Vimentin, E-Cadherin, Slug, Snail, ZEB1 and HMGA2. Overall, we speculate that microRNAs like miR-15b and miR-98 co-ordinate the regulation of the expression of E-cadherin, ZEB1 (TCF8), HMGA2 and SNAIL. Similarly, miR-29a, miR-221 and miR-23b in this network seem to regulate the expression of Vimentin and SLUG indirectly through Caspase 7, AP-1 (activator protein 1) and PAK (p21 protein activated kinase 2).

The results of the *in silico* analysis utilizing TargetScan revealed that many of the differentially regulated genes in our gene array can possibly be regulated by some of the differentially regulated microRNAs from the global microRNA profile (Table 3 & 4). Among the most targeted genes was Lifr, which drew our attention. It was found to be targeted by multiple microRNAs in our analysis, including miR-143, miR-30 family members, miR-140, miR-27b, miR-125a-5p, miR-128ab, and miR-342-3p. Interestingly, all these microRNAs were downregulated in the miRnome analysis, and correspondingly, Lifr was upregulated at the transcription and translational levels in parous ALDH positive MECs (Figure 3). We also looked at genes other than the ones differentially expressed in our gene array analysis Figure 5 and found that miR-125a targets many cell cycle genes, like Ccnd1, p21, and Cdk2, which may prove to be instrumental in unraveling the mechanism of parity-induced protection against breast cancer. Previously, miR-125a-5p was also reported to target Lin28, which is again very interesting in the context of stem cells. Here, we have demonstrated that parous ALDH positive MECs demonstrate a higher expression of Lin28 at the protein level than nulliparous ALDH positive MECs (Figure 3B).

Another highly targeted gene in our *in silico* analysis was Acvr1c or Alk7, a receptor involved in the nodal-activin pathway. It was found to be a putative target for let-7 family members, miR-26ab, miR-181 family, miR-150, miR-27b, miR-23ab, miR-425, miR-125a-5p, and miR-128ab. However, miR-125a-5p is the only microRNA that was found to target both Acvr1c and Lifr. Thus, we believe that Lifr and Acvr1c could be the major players in the modulation of ALDH positive MECs in response to pregnancy. Furthermore, because these genes are regulated by miR-125a-5p, it is possible that miR-125a-5p plays a key regulatory role in putative stem cells of MECs and is involved in parity-induced protection against breast cancer.

## Discussion

It is well known that early parity leads to a reduced risk of breast cancer. In this regard, numerous theories trying to explain the underlying mechanism have been postulated. However, at present, it has not been possible to completely unravel the mechanism behind this phenomenon. Previously, investigators have studied the differences between MECs of virgin and parous females of both humans and rodents. However, in this study, we have directed our efforts to look for the transcriptomic changes in the ALDH positive MEC population in virgin and parous rats. Our aim was to determine the underlying mechanism of parity-induced reduction in breast cancer risk in terms of changes introduced during the process of pregnancy in the ALDH positive MEC population. Therefore, in contrast to previous studies where changes in the entire mammary epithelium were analyzed, our study specifically scrutinized changes in putative stem cells of the MEC population.

To the best of our knowledge, this is the first report to describe changes in the global microRNA profiles between virgin and parous ALDH positive MECs. Our data identified the gene and microRNA signature induced by pregnancy in the ALDH positive MECs. Our *in vitro* data and *in silico* analysis led us to speculate about some of the possible phenomenon that can be attributed to the parity-induced reduction in breast cancer risk. We show here that ALDHpositive MECs of parous might differ from virgin in terms of regulation of the cell cycle, EMT processes, and tumor suppressors. The mammary gland is considered as an actively cycling tissue, which means that putative stem cells are under constant pressure to make decisions on cell cycle progression, proliferation, cell cycle arrest, and differentiation. There are several genes (Ccne1, Cdk2, E2f5 and Rbl1) in our dataset that demonstrate the possibility of an alteration in the regulation of cell cycle of ALDH positive MECs in response to pregnancy. Down regulation of microRNAs, like miR-28, miR-125a, and miR-503, could possibly lead to up regulation of p21 in parous ALDH positive MECs. This increase in p21 levels may lead to cell cycle arrest in the putative stem cells. This is further supported by our data, which show a down regulation of other significant cell cycle-associated genes, including Ccne1, Cdk2, E2f5, and Rbl1, in parous ALDH positive MECs. p21 is known to be suppressed in many human cancers; it was recently shown in a preclinical study that the up regulation of p21 leads to an anti-proliferative effect in breast cancer cell lines with cell cycle arrest in G1 phase [46]. Further, our cell proliferation data also indicates a higher proliferation rate in virgin ALDH positive MECs. These finding suggests that faster cycling ALDH positive MECs in virgin mammary glands would have a much greater chance of accumulating genetic mutations after carcinogenic insult compared to ALDH positive MECs in parous mammary gland, which may cycle at a much lower rate.

However, this speculation does not completely explain the phenomenon of parity-induced reduction in breast cancer risk. It has been observed in animal models that upon carcinogenic insult, the frequency of occurrence of neoplastic lesions is almost similar in both virgin and parous mammary glands [47]. This observation suggests that lesions in virgin glands are able to progress to breast cancer much more rapidly over time than in parous glands. Therefore, there must be additional factors other than cell cycle regulators that govern the progression of these neoplastic lesions. In this context, it is interesting to note that Lifr is highly upregulated in parous ALDH positive MECs. A recent report identified Lifr as a metastasis suppressor that executes its effects through the Hippo signaling pathway and prevents EMT [48]. In addition, we demonstrated the down regulation of Zeb2 and the up regulation of miR-218a in parous ALDH positive MECs. miR-218a in this study has been predicted to target Zeb2, which is another factor known to suppress EMT. Interestingly, miR-218 has been proved to regulate HMGB1 and suppress migration and invasion. This further supports the idea that ALDH positive MECs from parous mammary glands may not undergo EMT as efficiently as those from virgin glands. If this is indeed the case, then the regulatory environment of ALDH positive MECs in the parous gland does not favor progression of the disease. Thus, we speculate that in response to pregnancy, ALDHpositive MECs are altered in ways that not only favor slower cell cycle rates, but also lower efficiency of EMT like events, thus inhibiting progression of the disease. In this regard, our current data demonstrates that microRNAs likely play a key role in the regulation of these processes and therefore represent an important factor to be considered for their contribution to parity-induced reduction in breast cancer risk.

Another important point worth mentioning is Ppar*γ* (upregulated in parous ALDH positive MECs) is a highly targeted gene by most microRNAs in our dataset. Pparγ has been shown to promote the transcription of tumor-suppressor genes like Pten [49] and Brca1 [50]. With regards to stem cells, inhibition of Pparγ leads to the expansion of the stem cell pool as represented by the increased percentage of CK5+ and CD29+/CD24+ cells [51]. Interestingly, PPARγ*-*deficient mice also show an increased occurrence of hormone-dependent mammary cancers [51]. Taken together, we speculate that increased expression of Pparγ in parous ALDH positive MECs contributes to the regulation of stem cell fate determination, thus controlling the incidence of tumorigenesis. According to our *in silico* analysis, Ppar*γ* is likely regulated by microRNAs like let-7 family members, miR-30 family members, miR-27b, miR-23ab, miR-93, miR-25, miR-128ab, miR-320, and miR-135. In summary, the present study elucidates the differences in the ALDH positive MEC population in both parous and virgin glands. It was generally observed that there are changes at both the gene and microRNA levels in ALDH positive MECs of both groups, and these changes could contribute to reduced risk of breast cancer in parous mammary glands.

## Conclusion

Overall our data indicates that pregnancy alters microRNAs and genes in the ALDH positive MECs. These changes in the putative epithelial stem cell population render it resistant to mammary carcinogenesis. Also, our study brings forward a gene and microRNA signature specific to the ALDH positive MECs. These can serve as potential biomarkers and lead to the development of novel therapeutic targets for breast cancer.

## Electronic supplementary material

Additional file 1: Figure S1: Proliferation assay demonstrates higher cycling rates for virgin compared to parous ALDH positive MECs (n = 2; each group) were subjected to analysis after EdU treatment for 2 hours. A commercial kit was used to detect the incorporated EdU (molecular probes, Life Technologies). A) Representative confocal images (10X) for virgin and parous ALDH positive MECs showing the presence of cells at S-phase (green) of the cell cycle. B) Quantitative analysis to determine the percentage of proliferative cells in both the groups. (TIFF 107 KB)

## Abbreviations

MEC: Mammary epithelial cells
ALDH: Aldehyde dehydrogenase
DAPI: 4′ 6-diamidino-2-phenylindole
miRNA: microRNA
EMT: Epithelial Mesenchymal Transition.
