# Supplementary figures and images for: microRNA alterations in ALDH positive mammary epithelial cells: a crucial contributing factor towards breast cancer risk reduction in case of early pregnancy
Source: BMC Cancer. 2014 Aug 31;14:644. doi: 10.1186/1471-2407-14-644 (PMC4167510; doi:10.1186/1471-2407-14-644)

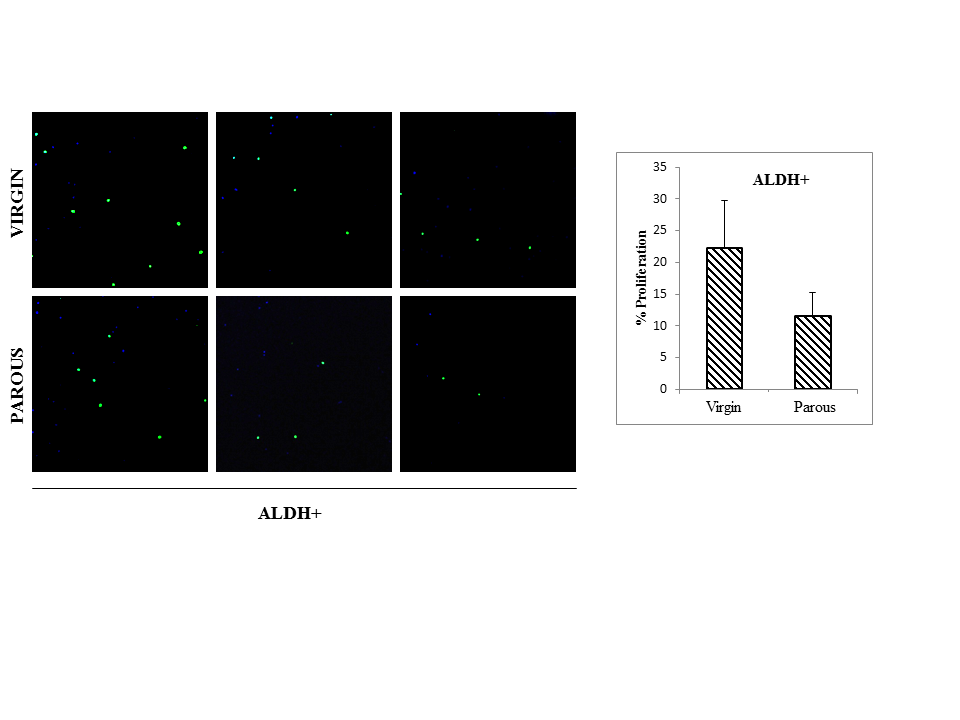

Supplement: Supplementary file 1 — Additional file 1: Figure S1: Proliferation assay demonstrates higher cycling rates for virgin compared to parous ALDH positive MECs (n = 2; each group) were subjected to analysis after EdU treatment for 2 hours. A commercial kit was used to detect the incorporated EdU (molecular probes, Life Technologies). A) Representative confocal images (10X) for virgin and parous ALDH positive MECs showing the presence of cells at S-phase (green) of the cell cycle. B) Quantitative analysis to determine the percentage of proliferative cells in both the groups. (TIFF 107 KB) [file 12885_2013_4842_MOESM1_ESM.tiff]
